# Cytokinin and auxin metabolism mediation of elevated [CO_**2**_]-enhanced shoot growth under different nitrogen conditions in perennial grass

**DOI:** 10.1093/hr/uhag025

**Published:** 2026-02-02

**Authors:** Ningli Fan, Qiuguo Li, Tian Hao, Danyi Wang, Peishuang Yang, Jingjin Yu, Zhimin Yang

**Affiliations:** College of Agro-grassland Science, Nanjing Agricultural University, Nanjing 210095, China; College of Agro-grassland Science, Nanjing Agricultural University, Nanjing 210095, China; College of Agro-grassland Science, Nanjing Agricultural University, Nanjing 210095, China; College of Agro-grassland Science, Nanjing Agricultural University, Nanjing 210095, China; College of Agro-grassland Science, Nanjing Agricultural University, Nanjing 210095, China; College of Agro-grassland Science, Nanjing Agricultural University, Nanjing 210095, China; College of Agro-grassland Science, Nanjing Agricultural University, Nanjing 210095, China

## Abstract

Elevated atmospheric [CO_2_] and nitrogen (N) availability are critical determinants of plant growth. This study investigated the underlying mechanisms of hormones in mediating elevated [CO_2_]-promoted shoot growth and leaf elongation under different N conditions in tall fescue (*Festuca arundinacea*). Plants were grown under low N (LN, 0.25 mM) and moderate N (MN, 4 mM) conditions. Subsequently, the plants from each N treatment were divided and immediately transferred to ambient (400 μmol mol^−1^) or elevated [CO_2_] (800 μmol mol ^−1^). Elevated [CO_2_] promoted plant growth under both LN and MN conditions through affecting cell division and cell elongation, with a more pronounced effect under MN supply levels. Elevated [CO_2_]-induced shoot growth and leaf elongation were associated with increased cytokinin level under LN and with enhanced cytokinin and auxin under MN conditions. Exogenous cytokinin inhibitor (lovastatin) and auxin inhibitor (2,3,5-triiodobenzoic acid) altered elevated [CO_2_]-enhanced growth in tall fescue regardless of N conditions. Elevation of [CO_2_]-enhanced growth by modulating cell growth-related genes *OsCycD2*, *OsPCNA*, and *OsEXPA10* was counteracted and reduced in *FaCKX11*-OE lines under LN and MN conditions, respectively. However, this enhancement was counteracted in *FaDAO-OE* lines under MN but not under LN conditions. These results demonstrated that elevated [CO_2_]-enhanced shoot growth in perennial grass species could be primarily mediated by cytokinin under LN conditions, while both cytokinin and auxin were involved in regulating elevated [CO_2_]-enhanced growth under MN conditions.

## Introduction

Atmospheric carbon dioxide concentration ([CO_2_]) has experienced a continuous increase from 280 to ~424 μmol mol^−1^ from industrialization to today [1]. Atmospheric [CO_2_] is expected to have doubled around the year 2100. As the main source of carbon for plant photosynthesis, [CO_2_] affects plant growth and development in nearly all physiological and biochemical processes [2].

Elevated [CO_2_]-induced growth promotion has been reported in numerous plant species [3]. For instance, in tall fescue (*Festuca arundinacea*), a C_3_ perennial grass, elevated [CO_2_] significantly promoted cell elongation and division, thereby leading to increases in shoot biomass and leaf length [4]. Enhanced shoot growth rate, stolon growth, and leaf growth were observed under elevated [CO_2_] in stoloniferous *Poa pratensis* [5], *Cynodon dactylon* [6], and *Agrostis stolonifera* [7]. Notably, elevated [CO_2_]-induced growth responsiveness was influenced by plant species, duration of CO_2_ exposure, growth stage, and environmental factors, including temperature conditions, water availability, and nutrient regimes [8–10].

Among environmental factors, nitrogen (N) was reported to particularly influence plant vegetative growth [11]. Furthermore, N availability influences the responses in plant growth to rising atmospheric [CO_2_], as these growth benefits derived from elevated [CO_2_] vary depending on its level and availability [12]. Elevated [CO_2_] was shown in our previous studies to promote leaf length and shoot height under moderate to high N supplies in tall fescue, whereas these positive effects were either absent or significantly reduced under low N conditions [4, 13]. A study in perennial grasslands showed that the response of plant biomass to elevated [CO_2_] was sensitive to N supply, with the stimulation of biomass under moderate N supply half that under high N supply [14]. In wheat (*Triticum aestivum*), elevated [CO_2_]-stimulated biomass was diminished to a suboptimal level under a limited N regime [15]. Elevated [CO_2_]-stimulated yield increases in rice (*Oryza sativa*) were contingent on N availability, with a greater response under high N conditions than under Low N conditions [16]. To date, the differential growth responsiveness to elevated [CO_2_] under varying N regimes have been well characterized. However, the mechanism driving these differential responses remains unclear and requires further investigation.

Plant hormones auxin and cytokinin are considered to be growth-promoting hormones, and their homeostasis was modulated by increased [CO_2_] [17]. For example, a study showed that the increased stolon length by elevated [CO_2_] in creeping bentgrass was attributed to the higher levels of indole-3-acetic acid (IAA) in the stolon [18]. Cytokinin and IAA levels were increased by elevated [CO_2_] in tall fescue plants, including roots and leaves [13, 19]. Elevated [CO_2_]-triggered accumulation of cytokinin and auxin in plants was due to the upregulation of their biosynthesis genes [13, 20]. These findings provide strong evidence that the endogenous cytokinin and auxin play positive roles in elevated [CO_2_]-enhanced plant growth. However, how they participate in the differential growth responsiveness to elevated [CO_2_] across varying N regimes is not well understood.

The endogenous contents of cytokinin and auxin are controlled not only through the synthesis pathway but also by the degradation process. The oxidation of IAA to its inactive form (2-oxindole-3-acetic acid, oxIAA) serves as the principal oxidative route for both IAA and IAA conjugates, and it constitutes the most poorly characterized step in its catabolism [21]. Cytokinin oxidase/dehydrogenase (*CKX*) gene families, which are key regulators of cytokinin levels, have been characterized in *O. sativa*, *Brassica rapa*, and *Malus domestica* [22–24]. *CKX* and *DAO* genes are generally involved in multiple physiological and developmental processes as well as environmental stimuli responses in many studies. However, the functions of *DAO*s and *CKX*s in regulating elevated atmospheric [CO_2_]-stimulated plant growth responses remain unclear. Furthermore, how elevated [CO_2_] differentially regulates plant growth under different N conditions through regulating *CKX*- and *DAO*-mediated hormone metabolism needs further investigation.

This study aimed (i) to determine the roles of cytokinin and auxin in elevated [CO_2_]-promoted growth under different N conditions through exogenous application of hormone inhibitors; (ii) to analyze cytokinin and auxin degradation metabolism-related gene expressions in elevated [CO_2_] exposure under different N conditions, subsequently identifying key responsive genes *FaCKX11* and *FaDAO* in tall fescue; and (iii) to investigate impacts of elevated [CO_2_] on transgenic lines alongside their wild counterparts under different N conditions. The elucidation of these mechanisms is essential for devising effective strategies that enhance grass growth performance and productivity under lower N input in scenarios with anticipated [CO_2_] enrichment.

## Results

### Effects of elevated [CO_2_] on endogenous hormone content under different N conditions

Our prior study demonstrated that elevated [CO_2_] differentially impacted tall fescue growth across N regimes, with the most significant effects observed under MN conditions [13]. To determine whether the differential effects were involved in endogenous hormone accumulation, we measured the endogenous level of IAA and isopentenyl adenosine (iPA). Under LN conditions, elevated [CO_2_] significantly increased iPA content, but had no effect on IAA content. Under MN conditions, both iPA and IAA contents were significantly increased by elevated [CO_2_] (Supplementary Data Fig. S1 [13]).

### Effects of exogenous hormone inhibitors on elevated [CO_2_]-enhanced growth under different N conditions

Initially, to screen the optimal hormone spray concentrations affecting tall fescue growth, plants cultured in half-strength Hoagland nutrient solution were sprayed with five concentrations of lovastatin (LOV) (0, 300, 500, 700, and 900 mg l^−1^) and four of 2,3,5-triiodobenzoic acid (TIBA) (0, 30, 45, and 60 mg l^−1^) in a growth chamber (Supplementary Data Fig. S2). The treatment with LOV at 500 mg l^−1^ caused the strongest shoot growth inhibition, reducing height by 16.6% compared with the control (0 mg l^−1^ LOV) after 35 days (Supplementary Data Fig. S2A), and was thus selected for further treatments. For TIBA treatment, 60 mg l^−1^ TIBA most effectively suppressed shoot height, decreasing it by 19.9% relative to the control after 21 days (Supplementary Data Fig. S2B), and was chosen for subsequent experiments.

**Figure 1:**
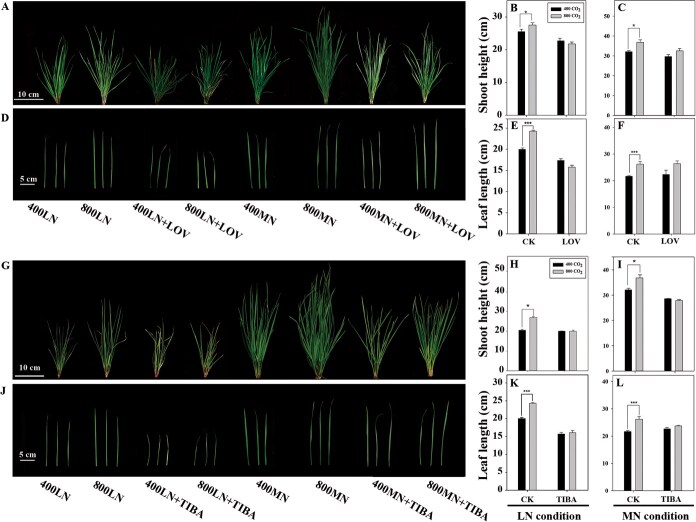
. Shoot growth of tall fescue treated with hormone inhibitors after CO_2_ and N treatments. (A–C) Phenotype and shoot height of tall fescue sprayed with LOV. (D–F) Leaf phenotype and length of tall fescue sprayed with LOV. (G–I) Shoot phenotype and height of tall fescue sprayed with TIBA. (J–L) Leaf phenotype and length of plants sprayed with TIBA. 400 CO_2_, ambient [CO_2_]; 800 CO_2_, elevated [CO_2_]. The asterisk (*) represents a significant difference between 400 CO_2_ and 800 CO_2_ under either LN or MN conditions (^*^P < 0.05, ^***^P < 0.001).

To elucidate the roles of cytokinin and auxin in elevated [CO_2_]-enhanced shoot growth, the cytokinin inhibitor (LOV) and the auxin inhibitor (TIBA) were applied to tall fescue plants grown under elevated [CO_2_] in two N regimes. Under LN conditions, shoot height of control plants (0 mg l^−1^ LOV or TIBA) was significantly improved due to elevated [CO_2_]; however, plant treatment with 500 mg l^−1^ of LOV or 60 mg l^−1^ of TIBA resulted in a diminished promotive effect of elevated [CO_2_] on shoot height. Similarly, under MN conditions, elevated [CO_2_] caused significant increases in shoot height of the control plants, but had no significant effects on shoot height in plants sprayed with either LOV or TIBA (Fig. 1A–C, G–I). Elevated [CO_2_] significantly enhanced leaf length in control plants regardless of N conditions. However, when plants were sprayed with either LOV or TIBA, no significant effect of elevated [CO_2_] was observed under both N conditions (Fig. 1D–F, J–L).

### Effects of elevated [CO_2_] on the expression of hormone degradation-related genes under different N conditions

To further investigate the roles of cytokinin and auxin in the varying effects of elevated [CO_2_] on growth under different N conditions, we analyzed the expression patterns of genes related to the degradation of cytokinin and auxin in this study, as effects of elevated [CO_2_] on hormone biosynthesis genes expression have been well documented.

This analysis was conducted at four time points: 0 h, 48 h, 14 days, and 21 days of treatment, through the quantitative reverse transcription polymerase chain reaction (qRT–PCR). Our previous study found that the auxin oxidase gene, *FaDAO*, was significantly downregulated by elevated [CO_2_] at 48 h and 21 days under both N conditions [13]. Since *FaDAO*-mediated auxin oxidation is a primary pathway for auxin degradation, we selected *FaDAO* for further analysis. For the cytokinin degradation pathway, four key degradation genes (*FaCKX1*, *FaCKX4*, *FaCKX8*, and *FaCKX11*) were analyzed (Supplementary Data Fig. S3). Under LN conditions, elevated [CO_2_] significantly upregulated *FaCKX1* expression at 14 days, but downregulated it at 21 days. Under MN conditions, elevated [CO_2_] significantly downregulated *FaCKX1* expression at 48 h and 14 days (Supplementary Data Fig. S3A). Elevated [CO_2_] had no significant effect on *FaCKX4* expression under LN conditions regardless of treatment duration. However, it significantly downregulated its expression at 48 h, 14 days, and 21 days under MN conditions (Supplementary Data Fig. S3B). The expression levels of *FaCKX8* were significantly inhibited due to elevated [CO_2_] at 48 h and 21 days under LN conditions; however, this inhibition was only significant during short time treatment (48 h) under MN conditions (Supplementary Data Fig. S3C). At 48 h and 21 days of treatment, elevated [CO_2_] significantly downregulated *FaCKX11* expression under both N levels, and at 14 days of treatment it also had significant repressive effect on *FaCKX11* expression under MN conditions (Supplementary Data Fig. S3D). Among the four genes, *FaCKX11* was selected for subsequent analysis as its expression patterns at 48 h and 21 days were consistent with cytokinin content changes after CO_2_ and N treatments.

### Enhanced cytokinin and auxin oxidation in *FaCKX11*- and *FaDAO*-transgenic lines

**Figure 2:**
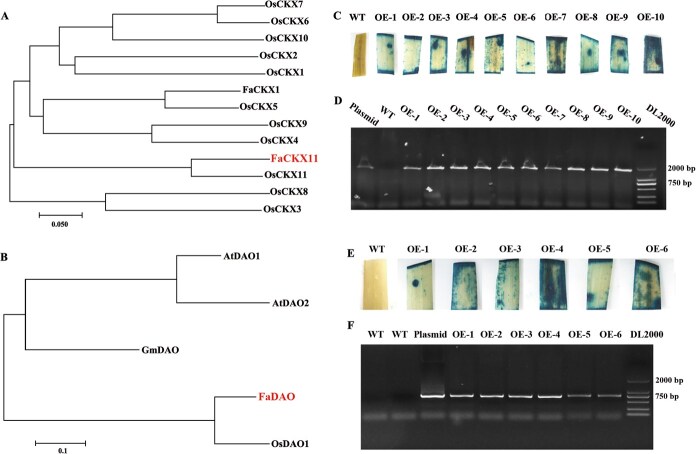
. Phylogenetic analysis and identification of positive transgenic lines. (A) Phylogenetic tree of CKX proteins from *F. arundinacea* and *O. sativa*. (B) Phylogenetic tree of DAO proteins from *F. arundinacea*, *O. sativa*, *Arabidopsis*, and *G. max*. (C) GUS staining of WT and *FaCKX11*-transgenic lines. (D) PCR amplification of CDS and partial vector sequence in *FaCKX11*-transgenic lines. (E) GUS staining of WT and *FaDAO*-transgenic lines. (F) PCR amplification of CDS and partial vector sequence in *DAO*-transgenic lines.

To investigate the biological functions of *FaCKX11* and *FaDAO*, as well as their roles in mediating elevated [CO_2_]-enhanced growth under different N conditions, the full-length *FaCKX11* and *FaDAO* sequences were cloned from the cDNA data of tall fescue. The open reading frame of *FaCKX11* was 1557 bp, encoding a protein of 518 amino acids. *FaDAO* contained 897 bp of open reading frame predicted to encode 298 amino acids. Phylogenetic analysis showed that FaCKX11 had the closest relationship to OsCKX11 among the 11 OsCKX family members in rice (Fig. 2A). Additionally, among DAO family members from *Arabidopsis*, rice, and soybean, the closest relationship was observed between FaDAO and OsDAO (Fig. 2B).

**Figure 3:**
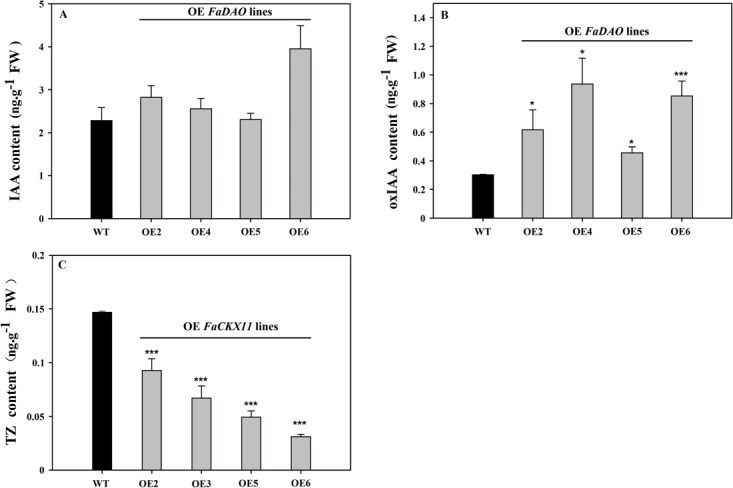
Effects of overexpression of *FaCKX11* or *FaDAO* on hormone content. (A) IAA content. (B) oxIAA content. (C) TZ content. The asterisk (*) represents a significant difference between WT and overexpression (*FaCKX11* or *FaDAO*) lines (^*^P < 0.05, ^***^P < 0.001).

To elucidate the functional roles of *FaCKX11* and *FaDAO*, the full-length gene sequences of *FaCKX11* and *FaDAO* were ectopically expressed in rice. A total of 10 independent T0 transgenic lines overexpressing *FaCKX11* (OE1–OE10) and 6 *FaDAO*-overexpressing lines (OE1–OE6) were successfully obtained (Fig. 2C–F). The GUS staining assay showed that leaves from transgenic lines displayed distinct blue coloration, whereas wild-type (WT) leaves remained colorless (Fig. 2C and E). In addition, the PCR amplification results revealed that a 2053-bp sequence, including 499 bp of vector and 1554 bp of *FaCKX11*, was amplified from the DNA template of transgenic lines overexpressing *FaCKX11* (Fig. 2D). The *FaDAO*-transgenic lines yielded a cloned segment containing partial vector sequence and *FaDAO*, whereas the DNA from WT plants did not amplify the target segments with the same primers (Fig. 2F). Overexpression of *FaDAO* did not significantly reduce free IAA levels in leaves; instead, it markedly enhanced the accumulation of oxIAA (Fig. 3A and B). Additionally, *FaCKX11*-transgenic lines OE1–OE4 were used for detecting thidiazuron (TZ) content and results revealed that overexpression of *FaCKX11* significantly decreased the content of TZ in leaves compared with that in WT plants (Fig. 3C).

### Effects of overexpression of *FaCKX11* and *FaDAO* on elevated [CO_2_]-enhanced growth under different N conditions

**Figure 4:**
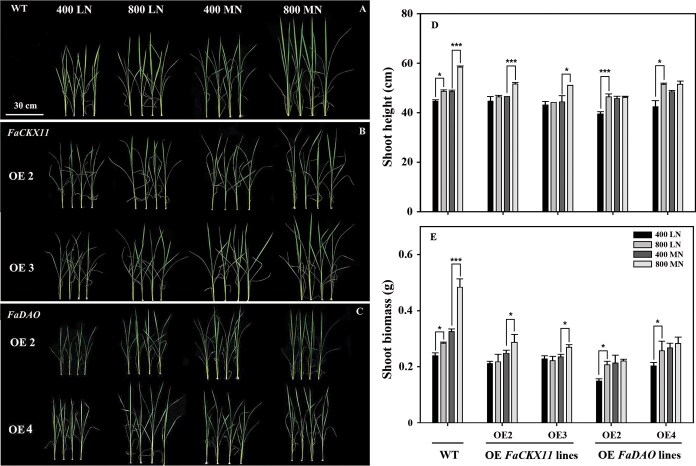
. Shoot growth of WT and overexpression lines (*FaCKX11* or *FaDAO*) after CO_2_ and N treatments. (A) WT. (B) Overexpression of *FaCKX11* lines. (C) Overexpression of *FaDAO* lines. (D) Shoot height of plants after CO_2_ and N treatment. (E) Shoot biomass of plants after CO_2_ and N treatment. 400, ambient [CO_2_]; 800, elevated [CO_2_]. The asterisk (*) represents a significant difference between ambient and elevated [CO_2_] under either LN or MN conditions (^*^P < 0.05, ^***^P < 0.001).

To investigate the roles of *FaCKX11* and *FaDAO* in mediating the growth enhancement associated with elevated [CO_2_] under different N conditions, we examined shoot height and biomass of both WT and transgenic plant lines exposed to two [CO_2_] levels across two N supply levels (Fig. 4). Under LN conditions, elevated [CO_2_] resulted in a significant increase in shoot height and biomass for WT plants, with increases of 9.2 and 18.9%, respectively (Fig. 4A, D, and  E). Similarly, two *FaDAO*-transgenic lines, OE2 and OE4, displayed significant increases in shoot height (17.6 and 21.4%) and biomass (40.3 and 19.6%) due to elevated [CO_2_] (Fig. 4C–E). In contrast, the stimulatory effects of elevated [CO_2_] were not observed in the *FaCKX11*-transgenic lines OE2 and OE3 (Fig. 4B, D, and  E). Under MN conditions, the enhancements in shoot height and biomass due to elevated [CO_2_] were even more pronounced in WT plants, with increases of 20.4 and 48.7%, respectively (Fig. 4A, D, and  E). For the *FaCKX11*-transgenic lines OE2 and OE3, elevated [CO_2_] also significantly increased shoot height by 11.1 and 14.7%, respectively, and shoot biomass by 15.9 and 14.8% (Fig. 4B, D, and  E). However, elevated [CO_2_] had no significant impact on shoot height or biomass of *FaDAO*-transgenic lines (Fig. 4C–E).

**Figure 5:**
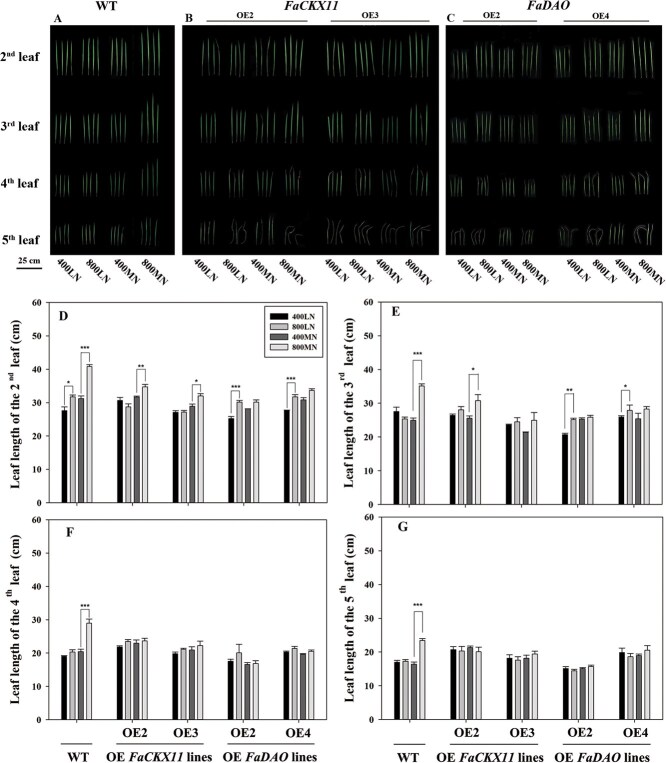
Leaf length of WT plants and overexpression lines (*FaCKX11* or *FaDAO*) after CO_2_ and N treatments. (A) WT. (B) Overexpression of *FaCKX11* lines. (C) Overexpression of *FaDAO* lines. (D) Second leaf. (E) Third leaf. (F) Fourth leaf. (G) Fifth leaf. 400, ambient [CO_2_]; 800, elevated [CO_2_]. The asterisk (*) represents a significant difference between ambient and elevated [CO_2_] under either LN or MN conditions (^*^P < 0.05, ^**^P < 0.01, ^***^P < 0.001).

To further examine whether the effects of *FaCKX11* and *FaDAO* on the elevated [CO_2_]-enhanced shoot growth under varying N supply levels were related to leaf elongation, leaf length of WT and transgenic rice was measured (Fig. 5). Under LN conditions, elevated [CO_2_] promoted the length of the second youngest leaf by 15.4% but had no effect on the other three leaves in WT plants (Fig. 5A, D–G). Additionally, elevated [CO_2_] enhanced the length of both the second and third leaves in *FaDAO*-transgenic lines (Fig. 5C–E). However, all of the tested leaves of *FaCKX11*-transgenic lines were not significantly altered by elevated [CO_2_] (Fig. 5B, D–G). Under MN conditions, the length of the second leaf was significantly increased by elevated [CO_2_] in both WT (30.7%) and *FaCKX11*-transgenic lines (9.6 and 10.8%) (Fig. 5A, B, and  D). Notably, no significant effect of elevated [CO_2_] was found on all tested leaves in *FaDAO*-transgenic lines (Fig. 5C, D–G).

### Endogenous hormone content in *FaCKX11*- and *FaDAO*-transgenic lines exposed to elevated [CO_2_] under different N conditions

**Figure 6:**
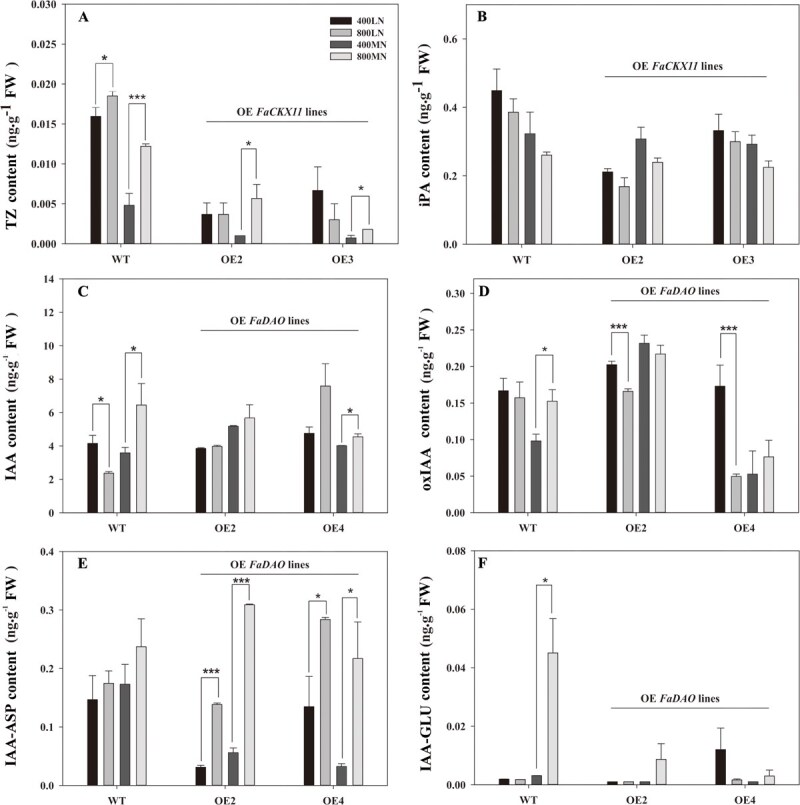
. Hormone content of WT and transgenic lines (overexpressing *FaCKX11* or *FaDAO*) after CO_2_ and N treatments. (A) TZ content. (B) iPA content. (C) IAA content. (D) oxIAA content. (E) IAA–ASP content. (F) IAA–GLU content. 400, ambient [CO_2_]; 800, elevated [CO_2_]. The asterisk (*) represents a significant difference between ambient and elevated [CO_2_] under either LN or MN conditions (^*^P < 0.05, ^***^P < 0.001).

To determine whether overexpressing *FaCKX11* and *FaDAO* altered the endogenous hormone metabolism in plants in response to elevated [CO_2_] under different N conditions, we quantified the final contents of cytokinin (TZ and iPA), auxin (IAA) and its metabolites [oxIAA, IAA–aspartate (IAA–ASP), and IAA–glutamate (IAA–GLU)] at 35 days of CO_2_ treatments under LN and MN conditions (Fig. 6). As shown in Fig. 6A, under LN conditions elevated [CO_2_] significantly increased TZ content in WT plants but not in *FaCKX11*-transgenic lines, which was consistent with the results in Fig. 4 showing that elevated [CO_2_] increased shoot height in WT but not in *FaCKX11*-transgenic lines. Under MN conditions, TZ content and shoot height in both WT and *FaCKX11*-transgenic lines was significantly increased by elevating [CO_2_] (Figs 4 and 6A). However, iPA content was not altered by elevated [CO_2_] under both N conditions (Fig. 6B).

The content of IAA in WT plants was significantly reduced by elevated [CO_2_] under LN conditions, whereas it increased under MN conditions. However, elevated [CO_2_] had no significant effects on IAA content in *FaDAO*-transgenic lines regardless of N conditions, except for an increase in OE4 under MN conditions (Fig. 6C). In WT plants, elevated [CO_2_] significantly increased oxIAA content under MN conditions but not under LN conditions, while it had no effect on IAA–ASP content regardless of N conditions. In *FaDAO*-transgenic lines, under LN conditions elevated [CO_2_] significantly decreased oxIAA content, but it led to a significant increase in IAA–ASP content. Under MN conditions, no significant change in oxIAA content was observed due to elevated [CO_2_], whereas IAA–ASP content showed a significant increase (Fig. 6D and E). Additionally, IAA–GLU content was not altered by elevated [CO_2_] in either WT or *FaDAO*-transgenic lines under LN conditions. However, elevated [CO_2_] resulted in a significant increase in IAA–ASP content in WT plants but not in transgenic lines on exposure to MN conditions (Fig. 6F).

### Expression analysis of growth-related genes in *FaCKX11*- and *FaDAO*-transgenic lines exposed to elevated [CO_2_] under different N conditions

**Figure 7:**
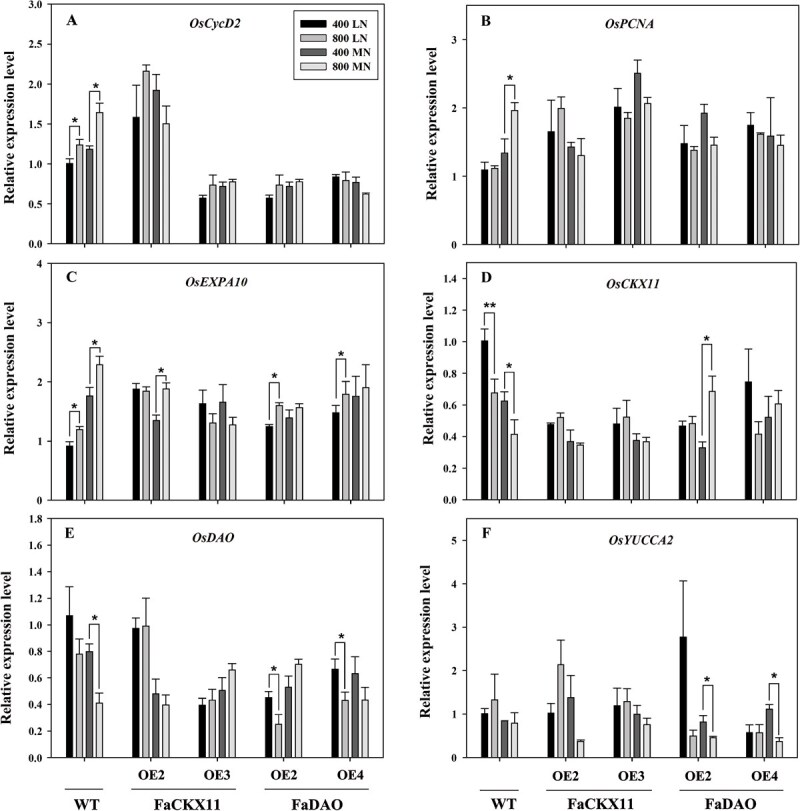
Gene expression levels of WT and overexpression lines (*FaCKX11* or *FaDAO*) after CO_2_ and N treatments. (A, B) Cell cycle-related genes *OsCycD2* and *OsPCNA*. (C) Cell elongation-related gene *OsEXPA10*. (D) Cytokinin degradation gene *OsCKX11*. (E) IAA degradation gene *OsDAO*. (F) IAA biosynthesis gene *OsYUCCA2*. 400, ambient [CO_2_]; 800, elevated [CO_2_]. The asterisk (*) represents a significant difference between ambient and elevated [CO_2_] under either LN or MN conditions (^*^P < 0.05).

To obtain further insights into the regulatory mechanism by which *FaCKX11* and *FaDAO* mediate the differential responses of shoot growth to elevated [CO_2_] under different N levels, we analyzed the expression patterns of key genes involved in cell division, cell elongation, and hormone metabolism by performing qRT–PCR (Fig. 7).

The expressions of two cell cycle-related genes (*OsCycD2* and *OsPCNA*) were significantly upregulated due to elevated [CO_2_] under MN conditions in WT plants; however, only *OsCycD2* exhibited enhanced expression under LN conditions. In both *FaCKX11*- and *FaDAO*-transgenic lines, elevated [CO_2_] had no significant effect on the expression levels of *OsCycD2* and *OsPCNA* under either LN or MN conditions (Fig. 7A and B). Expression of the cell elongation-related gene *OsEXPA10* was significantly upregulated by elevated [CO_2_] in WT plants under both N conditions, whereas the effect of elevated [CO_2_] was not detected in *FaCKX11*-transgenic lines except in OE2 under MN conditions. In contrast to *FaCKX11*-transgenic lines, elevated [CO_2_] significantly induced upregulation of *OsEXPA10* in *FaDAO*-transgenic lines under LN conditions (Fig. 7C). For hormone metabolism gene analysis, elevated [CO_2_] significantly decreased *OsCKX11* expression in WT under LN and MN conditions. However, this elevated [CO_2_]-responsive downregulation was not observed in all tested transgenic lines, except for *FaDAO*-OE2 under MN conditions (Fig. 7D). In addition, elevated [CO_2_] led to a significant decrease in *OsDAO* expression in WT plants under MN conditions but not under LN conditions. Conversely, elevated [CO_2_] significantly downregulated *OsDAO* expression in *FaDAO*-transgenic lines under LN conditions, whereas no such effect was observed under MN conditions. The expression level of *OsDAO* in *FaCKX11*-transgenic lines was also not changed by elevated [CO_2_] under the two N conditions (Fig. 7E). Auxin biosynthesis gene *OsYUCCA2* expression remained unaffected by elevated [CO_2_] in both WT and *FaCKX11*-transgenic lines under LN and MN conditions, whereas it was significantly downregulated by elevated [CO_2_] in *FaDAO*-transgenic lines under MN conditions (Fig. 7F).

## Discussion

Elevated [CO_2_] promotes plant growth, with more significant benefits observed under sufficient N conditions compared with insufficient conditions. This was observed in perennial grass species, including perennial ryegrass (*Lolium perenne*) [25] and tall fescue [13]. It has also been characterized in agronomic and horticultural crops, such as wheat [15] and tomato (*Solanum lycopersicum*) [12]. The variation in plant growth responses to elevated [CO_2_] under different N levels has been previously attributed to the carbon–nitrogen relationship in photosynthesis [26–29]. In our results, the content of iPA in leaves of tall fescue was increased by elevated [CO_2_] under LN and MN conditions, but the increases in IAA content in response to elevated [CO_2_] only occurred under MN conditions (Supplementary Data Fig. S1 [13]). Our previous research also found that IAA content was unaffected by elevated [CO_2_] under limited N supply but significantly upregulated under sufficient N conditions in both roots [19] and leaves [4] of tall fescue. However, a significantly increased IAA content in *Brassica napus* was detected in elevated [CO_2_] under LN availability [30]. The variability may be due to species differences, the degree of N deficiency, the way in which plants were fertilized, and sampling position [31]. In addition, the observation of increased cytokinin and auxin content due to elevated [CO_2_] under MN conditions suggests that elevated [CO_2_]-enhanced growth under MN conditions may be attributed to an increase in cytokinin and auxin concentrations (Supplementary Data Fig. S1 [13]). Furthermore, exogenous application of cytokinin inhibitor LOV and auxin inhibitor TIBA counteracted the growth promotion by elevated [CO_2_] under the two N regimes (Fig. 1). These findings provide strong evidence that cytokinin and auxin serve as a crucial driver in mediating the elevated [CO_2_]-induced shoot growth and leaf elongation with varying N supply levels in the perennial grass tall fescue.

Cytokinin oxidase governs endogenous cytokinin levels through the enhancement of cytokinin degradation [32]. Overexpression of *FaCKX11* caused significantly decreased TZ content in rice (Fig. 3C). The irreversible oxidation of IAA to oxIAA, catalyzed by DAO, is critical for maintaining local IAA homeostasis [33]. However, overexpression of *FaDAO* in rice led to no significant change in free IAA level but significantly increased oxIAA content (Fig. 3A and B). Similar results were also documented in *Arabidopsis thaliana* by Mellor *et al.* [34] and *O. sativa* by Zhao *et al.* [35]. Despite the fact that DAO enzyme is responsible for the oxidation of IAA, leading to its inactivation, cellular auxin homeostasis requires coordinated action of DAO with Gretchen Hagen 3 (GH3). A GH3 enzyme is a key component for conjugating free IAA with sugars, amino acids, and small peptides, or by forming methyl esters to maintain auxin at optimal levels for growth and development [34]. Therefore, the unchanged free IAA content in *DAO* overexpression lines might be due to the effective compensatory mechanism of IAA conjugates in controlling the pool of free hormones [35]. Further kinetic characterizations of *FaDAO* with different IAA conjugates, including IAA–ASP and IAA–GLU, are still needed. Nevertheless, these results further emphasize the critical roles of cytokinin and auxin in mediating the enhanced shoot growth associated with elevated [CO_2_].

To further investigate the roles of cytokinin and auxin in mediating elevating [CO_2_]-stimulated shoot growth under two different N supply levels, we utilized overexpression of *FaCKX11* and *FaDAO* to alter endogenous hormone metabolism (Figs 4 and 5). Under LN conditions, overexpression of *FaCKX11* completely inhibited the elevated [CO_2_]-stimulated growth promotion. Interestingly, similar growth enhancement was observed in both WT and transgenic lines overexpressing *FaDAO* under elevated [CO_2_]. The differential phenotypes of *FaCKX11* and *FaDAO* in response to elevated [CO_2_] under LN conditions might be explained by the inherent differences in their metabolic pathways, which lead to their distinct functions in response to environmental factors. Our data showed that elevated CO_2_ promoted growth by increasing cytokinin content under LN conditions. However, the *FaCKX11* gene is responsible for the irreversible degradation of cytokinins. Overexpression of *FaCKX11* caused an excessive decrease in cytokinin content, which offset the increase induced by elevated [CO_2_], leading to a loss of the growth promotion of elevated [CO_2_]. Further results on cytokinin content also supported this proposal (Fig. 6). In contrast, although *FaDAO* also encodes an enzyme for irreversible IAA oxidation, this represents only one branch of the auxin catabolic network. The reversible conjugation pathway can buffer changes in free IAA levels, thereby conferring greater resilience to environmental stimuli. Our results also showed that elevated [CO_2_] did not increase IAA content under LN conditions. A similarly unchanged IAA level was observed in the *FaDAO*-overexpressing lines under elevated [CO_2_], indicating that IAA accumulation and degradation via *FaDAO* are not the critical mechanisms for elevated [CO_2_] to promote growth under LN supply (Fig. 6). These results suggest that under LN conditions, cytokinin signaling is a more essential and nonredundant pathway for transducing the growth signal of elevated [CO_2_], whereas auxin signaling plays a more modulatory role. It is also plausible that the increased expression of *FaDAO* in transgenic lines did not sufficiently offset its elevated [CO_2_]-induced downregulation under N-deficient conditions, but complex crosstalk may exist between *FaDAO* and N signaling.

Under MN conditions, overexpression of *FaCKX11* or *FaDAO* could partially or even completely counteract the positive effects of elevated [CO_2_], suggesting that *FaCKX11* and *FaDAO* play critical roles in plant growth under elevated [CO_2_]. Previous studies have confirmed that elevated [CO_2_] increased levels of representative plant hormones such as auxins, gibberellins, and cytokinins [36]. Similar increases in the expression of related genes in the synthesis and transport pathway of auxin, cytokinin, and gibberellins by elevated [CO_2_] were reported [37]. High levels of cytokinin precursor content in *Arabidopsis* were observed under elevated [CO_2_]. However, while elevated [CO_2_] directly stimulates cytokinin biosynthesis, the subsequent accumulation of cytokinins could potentially lead to a feedback mechanism where the plant might increase cytokinin degradation via *CKX1*, *CKX4*, *CKX6*, and *CKX7* to maintain a balanced cytokinin level in *Arabidopsis* [20]. Therefore, in this study overexpression of *FaCKX11* or *FaDAO* might have triggered excessive feedback regulation, leading to a decrease in elevated [CO_2_]-induced cytokinin and auxin content, thereby diminishing the promotive effects of elevated [CO_2_] on transgenic lines. Since the expression levels of *FaCKX11* and *FaDAO* were suppressed by elevated [CO_2_], we cannot rule out the possibility that elevated [CO_2_] increases cytokinin and auxin content by directly modulating the hormone degradation pathways. Further investigation is needed to clarify the specific roles of high [CO_2_] in regulating these degradation pathway genes. In any case, our results propose that enhanced degradation metabolism generated by overexpressing *FaCKX11* and *FaDAO* directly or indirectly mediated elevated [CO_2_]-enhanced plant growth under MN conditions.

Although extensive research has confirmed that leaf elongation results from both cell division and cell elongation, the specific contributions of these developmental processes to leaf elongation mediated by *FaCKX11* and *FaDAO* have not been determined. In a previous study we demonstrated that increased cell number and longer cell length were the primary contributors to elevated [CO_2_]-induced leaf elongation [13]. Cell cycle-related genes play a pivotal role in regulating cell division [38]. The proliferating cell nuclear antigen (*PCNA*) gene is crucial for DNA replication and cell cycle regulation [39], while cyclin D2 (*CycD2*) promotes cell cycle activities and commitment to cell division via specifically accelerating G1 phase [40]. The shortening G1 phase leads to a reduction of meristem cell-cycle times in *CycD2*-transgenic plants [41]. In current results, the expression levels of *OsCycD2* and *OsPCNA* significantly increased in WT under elevated [CO_2_] conditions in MN environments. However, under LN conditions, only the expression of *OsCycD2* was upregulated (Fig. 7A and B). These results suggest that the increase in cell numbers due to elevated [CO_2_] may be attributed to cell division-related gene upregulation in leaves of tall fescue. Analogous to our finding, elevated [CO_2_] increased growth in *Dactylis glomerata* by shortening G1 phase and increasing the population of rapidly cycling cells [42]. In addition, proliferating PCNA accumulation was always higher in the high [CO_2_] condition [43]. In contrast, *FaCKX11* and *FaDAO*-transgenic lines were unable to upregulate expression of *OsCycD2* and *OsPCNA* in response to elevated [CO_2_], suggesting that *FaCKX11* and *FaDAO* mediate the elevated [CO_2_]-induced leaf growth under different N conditions by modulating the expression of cell cycle-related genes *OsCycD2* and *OsPCNA*, thereby affecting cell division. It is not excluded that other genes involved in cell division might be relevant to the mediating roles of *FaCKX11* and *FaDAO*.

In addition to cell cycle-related genes, researchers have characterized several cell wall extension-related genes, among them expansin genes [44, 45]. Expansins regulate cell extensibility and leaf elongation [46, 47]. In the current study, *OsEXPA10* expressions were significantly increased by elevated [CO_2_] in WT plants regardless of N conditions (Fig. 7C). This pattern aligns with [CO_2_]-induced cell elongation observed in tall fescue leaves [13]. Interestingly, consistent with elevated [CO_2_]-enhanced growth in *FaDAO*-transgenic lines under LN conditions, elevated [CO_2_] also induced the upregulation of *OsEXPA10* in *FaDAO*-transgenic lines under the same conditions. In a previous study *OsEXPA10* was identified as a key regulator of cell elongation in rice [48]. Therefore, it is tempting to speculate that *OsEXPA10* might be a critical mediator allowing elevated [CO_2_] to positively impact the growth of *FaDAO*-transgenic lines under LN conditions. However, in our expression analysis, *OsEXPA10* expression was not altered by elevated [CO_2_] in *FaCKX11*-transgenic lines under both N conditions. Notably, the growth of these transgenic lines was still promoted by elevated [CO_2_] under MN supply levels, although at a lower level than WT, suggesting that *OsEXPA10* is not the sole gene involved in *FaCKX11* mediating elevated [CO_2_]-induced cell elongation under sufficient N conditions. The expansin family comprises multiple genes, with each member playing diverse functions in affecting leaf elongation in tall fescue [49]. Future research aims to identify other family members that may also mediate plant growth under elevated [CO_2_] conditions across varying N regimes.

## Conclusions

**Figure 8:**
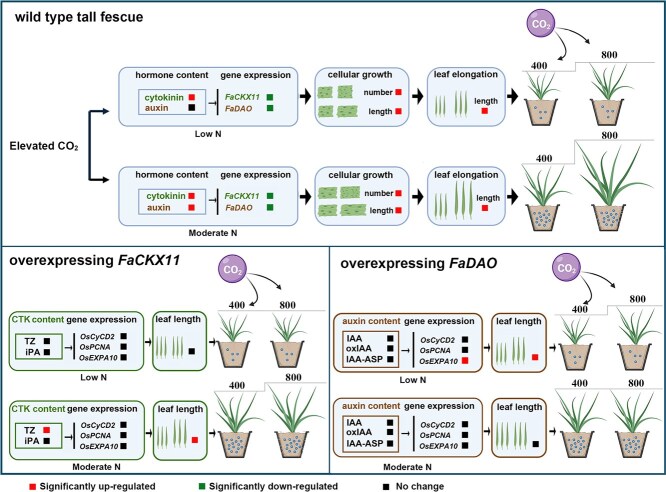
. A proposed model for cytokinin- and auxin-mediated differential regulation of shoot growth and leaf elongation in plants exposed to elevated [CO_2_] under different N conditions, associated with altered expression of *FaCKX11* and *FaDAO*.

In summary, rising levels of CO_2_ significantly enhanced shoot growth and leaf elongation via promoting cell division and elongation in tall fescue under N-deficient and -adequate conditions, with the stimulatory effect being more evident under adequate N supply. The elevated [CO_2_]-induced differential effects corresponded to higher concentrations of cytokinin under LN conditions and increased levels of both cytokinin and auxin under MN conditions. This enhancement of elevated [CO_2_] was counteracted in lines that overexpressed *FaCKX11*, while it still occurred in *FaDAO*-transgenic lines under LN conditions. However, the positive impact of elevated [CO_2_] on growth was diminished in *FaCKX11*- and *FaDAO*-transgenic lines, indicating a distinction in the hormonal response. Overall, cytokinin and auxin metabolism mediated the elevated [CO_2_]-enhanced shoot growth by modulating cell cycle-related genes (*OsCycD2* and *OsPCNA*) and a cell elongation gene (*OsEXPA10*) under different N conditions. These findings highlight the complex interplay between environmental factors and plant hormonal responses, suggesting that understanding these mechanisms is vital for improving plant resilience and productivity in climate change conditions. Future study should focus on further exploring the molecular pathways regulated by elevated CO_2_, particularly the interactions between cytokinin, auxin, and other hormones under varying nutrient conditions. Additionally, conducting long-term field studies to explore the practical implications of these findings in diverse agricultural settings could provide valuable insights on how to optimize plant growth and yield in a future with rising atmospheric CO_2_ concentration (Fig. 8).

## Materials and methods

### Experiment 1: the roles of cytokinin and auxin in elevated [CO_2_]-enhanced shoot growth under different N conditions via hormone inhibitor application

#### Plant materials and growth conditions

Tall fescue (*F. arundinacea*) cultivar ‘Barlexas’ was used in this study. Seeds were germinated by uniformly distributing them on moist filter paper in glass dishes for 10 days. After germination, seedlings were cultivated with half-strength Hoagland nutrient solution in black pots (40 cm × 30 cm × 14 cm). Plant growth conditions in an environmentally controlled growth chamber (Model XBQH-1, Xubang, Shandong, China) were set as described by Fan *et al.* [13].

#### Experimental treatments

After 21 days of establishment, tall fescue plants with uniform plant height and tiller number were selected for CO_2_ and N treatments. These plants were planted in half-strength Hoagland solution with either LN (0.25 mM NO_3_^−^) or MN (4 mM NO_3_^−^) supplement. The nutrient solution was replenished weekly. A total of four replicates were established per N treatment, and each replicate comprised 15 individual plants. Subsequently, these plants were placed in growth chambers set at either ambient [CO_2_] (400 ± 10 μmol mol^−1^) or elevated [CO_2_] (800 ± 10 μmol mol^−1^). Each CO_2_ treatment was performed in two independent growth chambers, with each chamber including two N regimes [4]. A split-plot design with main plots of [CO_2_] and subplots of N levels was used. All treatments were maintained for 21 days in environmentally controlled growth chambers under the aforementioned controlled conditions.

For exogenous spray treatments in tall fescue, LOV and TIBA were used as exogenous cytokinin and auxin inhibitors, respectively [50, 51]. To screen for the optimal spray concentration suitable for effecting tall fescue growth, 21-day-old plants maintained in half-strength Hoagland solution were sprayed with LOV (0, 300, 500, 700, and 900 mg l^−1^) or TIBA (0, 30, 45, and 60 mg l^−1^) in a regimen of 7-day intervals over 35 and 21 days, respectively. Spray treatments were performed at 9:00 a.m. with a handheld sprayer, with the operator maintaining a distance of 20 cm during spraying. Each treatment comprised four biological replicates, with each replicate containing 15 individual plants. Shoot height of foliar-sprayed plants was recorded at weekly intervals. According to shoot height analysis, the optimal LOV or TIBA concentration for subsequent treatments was determined as the lowest concentration that induced a significant effect on shoot height.

After foliar application on 21-day-old tall fescue at predetermined optimal concentrations of LOV or TIBA, the plants were transferred to LN and MN conditions, followed by immediate transfer into growth chambers set at ambient or elevated [CO_2_] conditions. Each treatment had four repeats, and each repeat had 15 individual plants. Spray applications and nutrient solution renewal were performed weekly. Growth-related parameters were measured after treatments for 35 days.

#### Measurements of growth-related parameters and hormone contents

The measurements of shoot height, shoot biomass, and leaf length were conducted according to Fan *et al.* [13]. Liquid chromatography–triple quadrupole mass spectrometry (LC–MS/MS) was employed based on the method previously reported by Li *et al.* [52] for iPA extraction. The quantitative analysis of iPA was conducted by Nanjing Innovation Biotechnology Co., Ltd.

#### Gene expression analysis

Total RNA was extracted from leaves (50 mg) sampled at 0 h, 48 h, 14 days, and 21 days from tall fescue subjected to CO_2_ and N treatments. The reverse transcription of RNA was performed as described in Fan *et al.* [19]. The expression levels of *FaCKX1*, *FaCKX4*, *FaCKX8*, and *FaCKX11* were determined using qRT–PCR on a LightCycler 480 II machine (Roche Diagnostic, Rotkreuz, Switzerland). The qRT–PCR amplification conditions followed the description in Fan *et al.* [13]. Primers were also designed using an online Primer3 tool (Supplementary Data Table S1). Four biological replicates were performed within an experiment. The relative expression of target genes was determined employing the 2^−ΔΔCT^ method, which was standardized using the internal gene *FaTubulin* (Supplementary Data Table S1).

#### Statistical analyses

All statistics were analyzed using SPSS 13.0 software (SPSS Inc., Chicago, IL, USA). Multiple group comparisons of shoot height across gradient concentrations of exogenous treatments were conducted by one-way ANOVA, with significance determined at *P* < 0.05. For pairwise comparisons of shoot height, leaf length, gene expression level, and hormone content between two [CO_2_] treatments, two-sided Student’s *t*-test was employed. Statistical data were expressed as mean ± standard error (SE).

### Experiment 2: the roles of cytokinin and auxin in elevated [CO_2_]-enhanced shoot growth under different N conditions through overexpressing FaCKX11 and FaDAO in rice

#### Plant materials and growth conditions

Rice (*O. sativa*) was used for genetic transformation owing to its robust transformation system and close phylogenetic relationship with tall fescue. This strategy has been successfully employed in prior research on tall fescue [53]. Seeds of WT rice (‘Nipponbare’) and transgenic lines overexpressing *FaCKX11* or *FaDAO* were germinated on a half-strength Murashige and Skoog agar medium and incubated at 30°C for 7 days under controlled conditions.

#### Experimental treatments

Rice plants, including both WT and transgenic lines with uniform size, were subjected to half-strength Hoagland solution containing either 0.25 or 4 mM NO_3_^−^, representing LN and MN treatments, respectively. Subsequently, plants were exposed to CO_2_ treatments in growth chambers maintained at either 400 ± 10 or 800 ± 10 μmol mol^−1^ [CO_2_], representing ambient and elevated [CO_2_] treatments, respectively. The growth chambers were set at 30/25°C (day/night temperature), 70% relative humidity, and a 14-h photoperiod with 600 μmol m^−2^ s^−1^ photosynthetically active radiation at canopy level [53]. Two independent chambers were used for each CO_2_ level. Within each chamber, two N regimes were applied [4]. A total of four replicates were established per treatment combination, with each replicate consisting of 15 individual plants.

#### Measurement of growth-related parameters and hormone content

Measurements of shoot height, shoot biomass, and leaf length were conducted according to the methods in the Experiment 1 section Measurements of growth-related parameters and hormone contents. The extraction and quantification of phytohormones, including TZ, iPA, IAA, oxIAA, IAA–ASP conjugate, and IAA–GLU conjugate, was conducted according to the methods in the Experiment 1 section Measurements of growth-related parameters and hormone contents. Leaf samples were collected from 14-day-old WT rice and transgenic lines overexpressing *FaCKX11* or *FaDAO* grown in Kimura B nutrient solution in a growth chamber without additional treatments, or exposed to CO_2_ and N treatments for 35 days.

#### Identification and phylogenetic analysis of FaCKX11 and FaDAO

Partial nucleic acid sequences of *FaCKX11* and *FaDAO* were acquired from a tall fescue transcriptome database (NCBI Sequence Read Archive: PRJNA1271684). The protein sequences of rice OsCKX1 (LOC4325668), OsCKX2 (LOC4327333), OsCKX3 (LOC4348932), OsCKX4 (LOC4326515), OsCKX5 (LOC4327887), OsCKX6 (LOC107275892), OsCKX7 (LOC107275724), OsCKX8 (LOC107275925), OsCKX9 (LOC4338605), OsCKX10 (LOC107276418), and OsCKX11 (LOC4345764); rice OsDAO (Os04g0475600); soybean (*Glycine max*) GmDAO (XP_003517110.2); and *Arabidopsis* AtDAO1 (AT1G14130) and AtDAO2 (AT1G14120) were retrieved from the RiceData database (www.ricedata.cn), NCBI, and TAIR, respectively. The phylogenetic tree of these amino acid sequences was reconstructed with MEGA7.0 using the UPGMA method.

#### Genetic transformation of rice

To construct *FaCKX11* and *FaDAO* overexpression (OE) rice lines, their full-length CDSs were amplified from tall fescue cDNA with specific primers for these genes (Supplementary Data Table S2). The CDSs of *FaCKX11* and *FaDAO* were ligated into the pENTR™1A plasmid, linearized, and finally recombined into pVT1269 through LR recombination. The recombination constructs were transformed into *Agrobacterium tumefaciens* strain EHA105. Then, rice was transformed by infecting calli generated from seeds with the recombinant *Agrobacterium*, and these infected calli were selected with 400 mg l^−1^ hygromycin, following a previously described method [54, 55]. Finally, the resistant transformants were subjected to organogenesis medium with 2 mg l^−1^ kinetin. To confirm successful transformation, we performed GUS staining on young leaf samples from regenerated plants for *FaCKX11* or *FaDAO* overexpression according to the description in Zhang *et al.* [53]. The genomic integration of the target gene was also confirmed via PCR using primers containing both vector sequence and gene-specific sequences. Primer information is provided in Supplementary Data Table S3. Seeds from T3 transgenic lines were used for experimental treatments.

#### Gene expression analysis

qRT–PCR was employed to analyze the transcriptional level of *OsCycD-2*, *OsPCNA*, *OsEXPA10*, *OsCKX11*, *OsDAO*, and *OsYUCCA2* in WT, *FaCKX11* overexpression lines, and *FaDAO* overexpression lines, after treatments with CO_2_ and N for 35 days. Target gene primer and reference gene *OsActin* information are provided in Supplementary Data Table S4. The experiment included four biological replicates. All other procedures are described in the Experiment 1 section Gene expression analysis.

#### Statistical analyses

All statistical analysis software, methods, and data presentation were identical to those described in the Experiment 1 section Statistical analyses.

## Supplementary Material

Web_Material_uhag025

## Data Availability

All the data generated or analyzed underlying this article are available within the article and its supplementary data files.
