# Supplementary material for: Cytokinin and auxin metabolism mediation of elevated [CO2]-enhanced shoot growth under different nitrogen conditions in perennial grass
Source: Hortic Res. 2026 Feb 2;13(5):uhag025. doi: 10.1093/hr/uhag025 (PMC13148157; doi:10.1093/hr/uhag025)
Supplement: Web_Material_uhag025 [file web_material_uhag025.zip › Supplementary Table.docx]

**Table S1 Primers used for gene expression analysis in tall fescue**

| Genes | Forward primers | Reverse primers |
| --- | --- | --- |
| *FaTublin*  *FaCKX1*  *FaCKX4*  *FaCKX8*  *FaCKX11* | ATGCTTTCGTCTTATGCCC  GTCGTGAAGATGGAGTCCCT  GATCGAGAGCACAGAGGGAG  GCACGATTTCCAGCAAGACA  TAATGATGGCGGCTGATTCG | GTCTCATGCAGCACGTTGAT  GTCTCATGCAGCACGTTGAT  CAGTTGTTGAGAATGCCCGT  CACATTGAGCCATGGGTGAG  GCACCTAACAGCGACCCTAT |

**Table S2 Primers used for amplifying *FaCKX11* and *FaDAO***

| Genes | Forward primers | Reverse primers |
| --- | --- | --- |
| *FaCKX11*  *FaDAO* | TGCCCAAGATGATGCTCGCGTACAT ACAGCGCAGATGGTGGGGAT | ACCGACGTGTTAGGGTCCCACT GCTACGTCACGCCGCCATCC |

**Table S3 Primers used for identification of transgenic lines**

| Genes | Forward primers | Reverse primers |
| --- | --- | --- |
| *FaCKX11*  *FaDAO* | CCCTGTTGTTTGGTGTTACTT  GGGCGGTCGTTCATTCGTT | ACCGACGTGTTAGGGTCCCACT  AATCCGCTCTACGAGGCA |

**Table S4 Primers used for gene expression analysis in rice**

| Genes | Forward primers | Reverse primers |
| --- | --- | --- |
| *OsPCNA*  *OsCycD2*  *OsEXPA10*  *OsCKX11*  *OsDAO*  *OsActin* | TGGACAGGTTATCACCATTGGT  TAGAGATGCAGGAACCGGTG  TGCGTGGATGACCTCAAGTA  TCTACCCCATGCTCAAGTCC  TTTATTACGCGGAGTGCTCA  TGGACAGGTTATCACCATTGGT | CCGCAGCTTCCATTCCTATG  TGCGACAATCACGACTTCAT  ACACCACCATCACGAAGACA  CGTCAATGATTGCGTTGTTC  CTCCTCCAGCCAAACAAAAG  CCGCAGCTTCCATTCCTATG |
