# Supplementary material for: Cytokinin and auxin metabolism mediation of elevated [CO2]-enhanced shoot growth under different nitrogen conditions in perennial grass
Source: Hortic Res. 2026 Feb 2;13(5):uhag025. doi: 10.1093/hr/uhag025 (PMC13148157; doi:10.1093/hr/uhag025)
Supplement: Web_Material_uhag025 [file web_material_uhag025.zip › Supplementray Figure.docx]

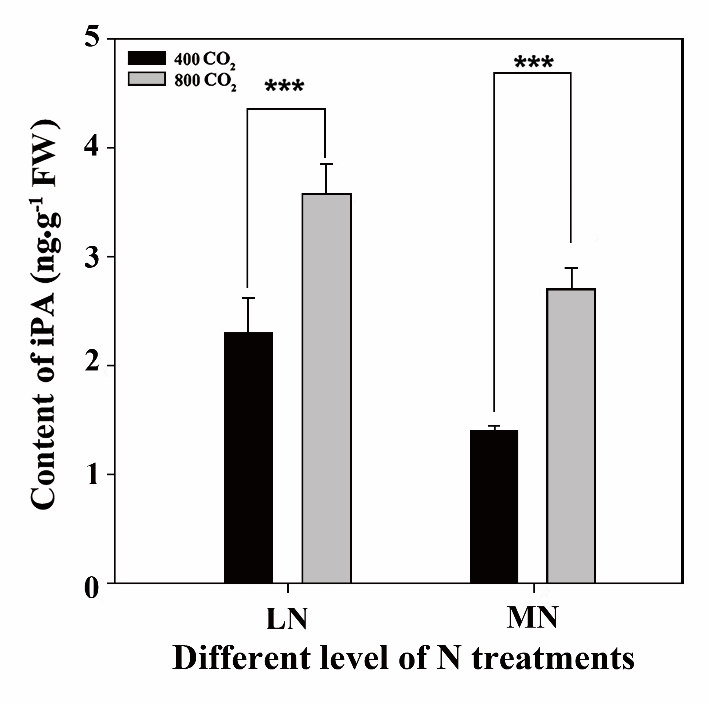


**Figure S1**. Cytokinin content in tall fescue after CO_2_ and N treatments. 400 CO_2_, ambient [CO_2_]; 800 CO_2_, elevated [CO_2_]; LN, low N; MN, moderate N. * represents a significant difference between ambient and elevated [CO_2_] under either LN or MN conditions, respectively (^***^ *P* < 0.001).


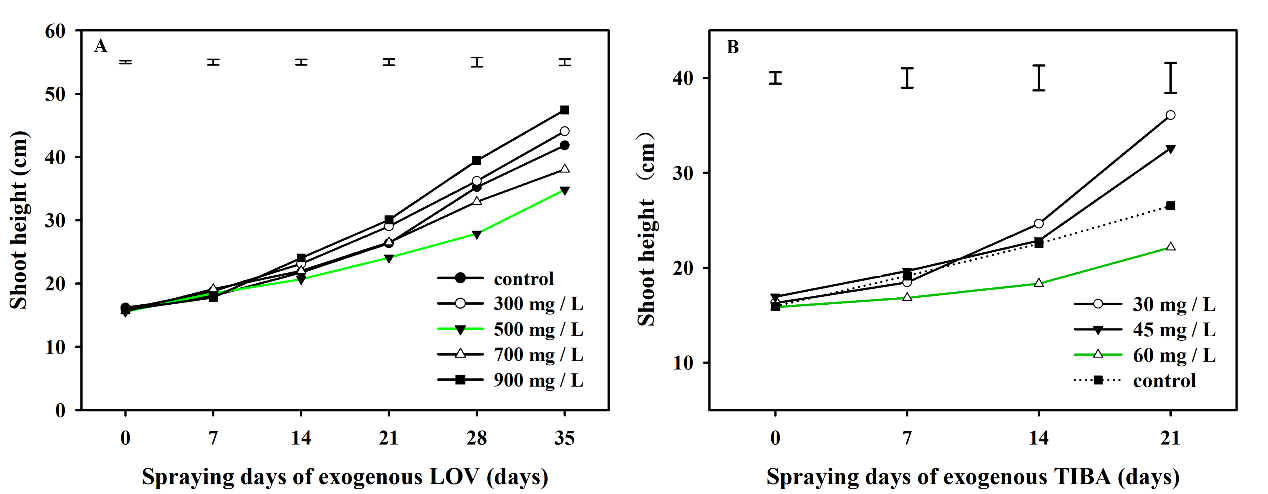


**Figure S2**. Effects of exogenous application of hormones inhibitors on shoot height in tall fescue. A, Application of five concentrations of cytokinin inhibitor LOV. B, Application of four concentration of auxin inhibitor TIBA. Based on LSD test (*P* < 0.05), significant differences among treatments are indicated by vertical bars.


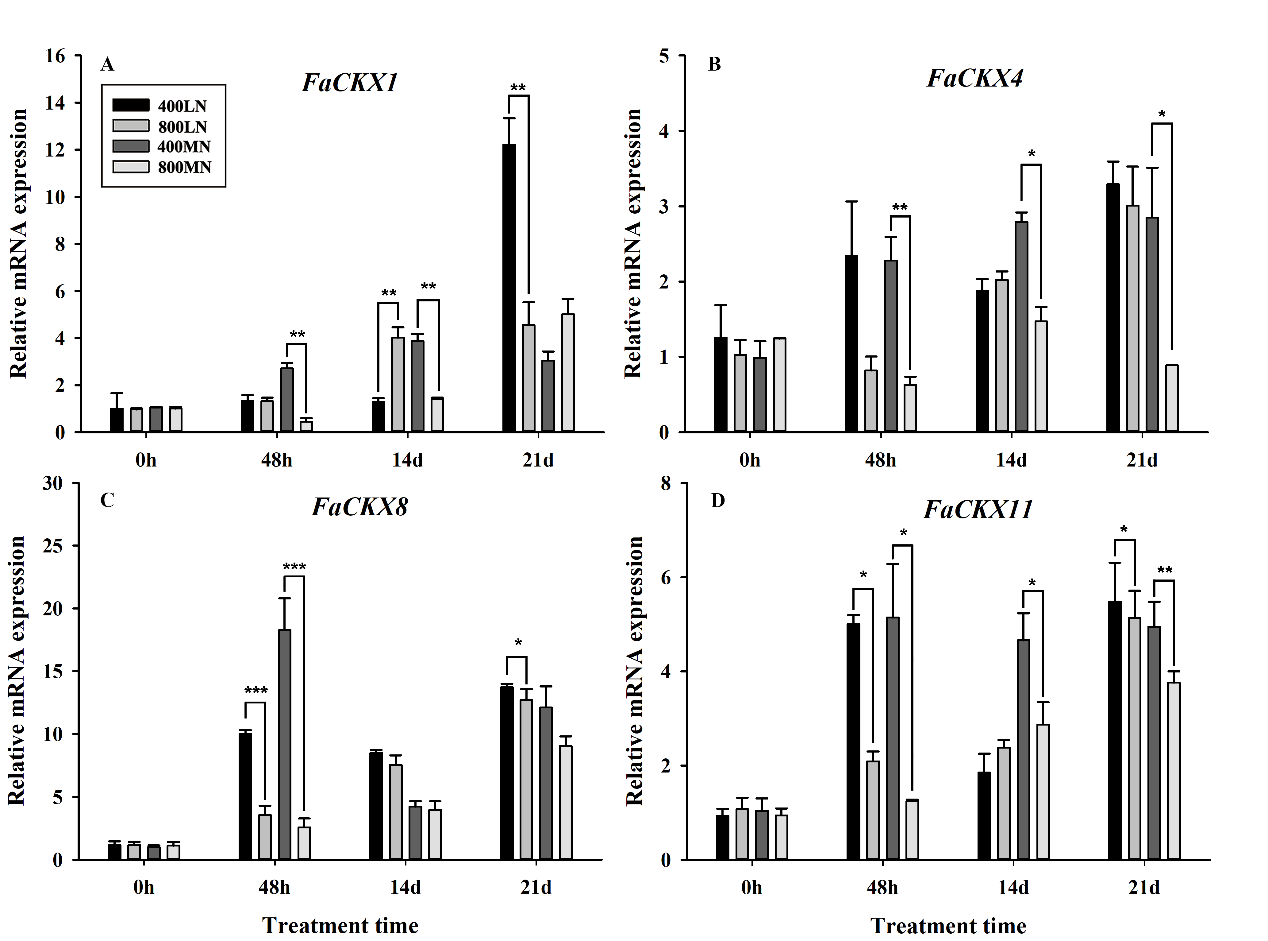


**Figure S3**. Expression levels of cytokinin degradation genes after CO_2_ and N treatments. 400, ambient [CO_2_]; 800, elevated [CO_2_]; LN, low N; MN, moderate N. * represents a significant difference between two [CO_2_] treatments under LN and MN conditions, respectively (^*^ *P* < 0.05, ^**^ *P* < 0.01, ^***^ *P* < 0.001).
